# The role of ALBI score in patients treated with stereotactic body radiotherapy for locally advanced primary liver tumors: a pooled analysis of two prospective studies

**DOI:** 10.3389/fonc.2024.1427332

**Published:** 2024-10-03

**Authors:** Eleni Gkika, Gianluca Radicioni, Alexandra Eichhorst, Simon Kirste, Tanja Sprave, Nils Henrik Nicolay, Stefan Fichtner-Feigl, Robert Thimme, Rolf Wiehle, Thomas B. Brunner, Anca-Ligia Grosu

**Affiliations:** ^1^ Department of Radiation Oncology, Medical Center - University of Freiburg, Freiburg, Germany; ^2^ German Cancer Consortium (DKTK) Partner Site Freiburg, Freiburg, Germany; ^3^ Faculty of Medicine, University of Freiburg, Freiburg, Germany; ^4^ Department of Radiation Oncology, University Clinic Bonn - University of Bonn, Bonn, Germany; ^5^ Department of Radiation Oncology, University Medical Center Leipzig, Leipzig, Germany; ^6^ Department of General and Visceral Surgery, Medical Center - University of Freiburg, Freiburg, Germany; ^7^ Department of Medicine II, Gastroenterology, Hepatology, Endocrinology and Infectious Diseases, Medical Center - University of Freiburg, Freiburg, Germany; ^8^ Department of Radiation Oncology, University Medical Center Graz, Graz, Austria

**Keywords:** HCC, CCC, SBRT, hepatocellular carcinoma, cholangiocarcinoma, radiotherapy

## Abstract

**Introduction:**

To evaluate the outcomes after stereotactic body radiotherapy (SBRT) for locally advanced primary liver cancer.

**Materials and methods:**

Patients with locally advanced liver cancer unsuitable for other loco-regional treatments were treated with SBRT with 50–60 Gy in 3–12 fractions in two consecutive prospective trials.

**Results:**

A total of 83 patients were included, of whom 14 were excluded, leaving 69 evaluable patients with 74 treated lesions. A total of 50 patients had hepatocellular carcinoma (HCC), and 11 patients had cholangiocarcinoma (CCC). Approximately 76% had a Child-Pugh (CP) score of A, while 54% had an albumin–bilirubin (ALBI) score of 1. With a median follow-up of 29 months, the median overall survival (OS) was 11 months, and the progression-free survival (PFS) was 18 months. The ALBI score was an important predictor of overall survival (HR 2.094, p = 0.001), which remained significant also in the multivariate analysis. Patients with an ALBI grade of ≥1 had an OS of 4 months versus 23 months in patients with an ALBI grade of 1 (p ≤ 0.001). The local control at 1 and 2 years was 91%. Thirteen patients developed grade ≥ 3 toxicities, of whom nine patients experienced liver toxicities. Patients with a higher ALBI score had a high risk for developing hepatic failure (OR 6.136, p = 0.006).

**Discussion:**

SBRT is a very effective treatment with low toxicity and should be considered as a local treatment option in patients with HCC and CCC. Patients with a higher ALBI grade are at risk for developing toxicities after SBRT and have a significantly lower survival rate.

## Introduction

Liver cancer has a very poor prognosis. Approximately 90% of the cases globally are diagnosed with hepatocellular carcinoma (HCC), which is the most common primary liver tumor, while other primary tumors including cholangiocarcinoma (CCC) are rare. The only curative treatment options are either surgery or transplantation and, in some cases, radiofrequency ablation (RFA). However, fewer than 30% of patients are eligible for these treatments (1, 2). Other local treatment options include trans-arterial chemoembolization (TACE) especially for HCC, selective internal radiation therapy (SIRT), high-dose-rate (HDR) brachytherapy, or stereotactic body radiotherapy (SBRT). The immune microenvironment plays a significant role in the development and progression of HCC, while inflamed and non-inflamed classes of HCC and genomic signatures have been associated with response to immune checkpoint inhibitors (3). In the past years, there has been increasing evidence of the role of circular RNAs in the modulation of proliferation and metastases of cancer cells, while epithelial–mesenchymal transition (EMT) enhances metastasis and invasion of tumor cells and can trigger resistance to therapy (4). Additionally, the tumor microenvironment remolds, and the acceleration of immunotherapy and vaccines can be provided by peptide nanoparticles (4, 5).

Due to the technological advances of the past decades, SBRT has been proven to be a very efficacious local treatment with high rates of local control ranging from 75% to 100% and can be used safely with low rates of toxicity in both HCC and CCC. SBRT leads to similar local control rates compared to RFA or TACE (6–9). Thus, according to the National Comprehensive Cancer Network (NCCN) guidelines, SBRT is not only an alternative for HCC patients ineligible for other local treatments but also a treatment option *a priori* equal to other local treatments, such as ablation or arterially directed therapies. Nevertheless, due to the lack of randomized trials, SBRT is not yet included in the current Barcelona Clinic Liver Cancer (BCLC) classification. However, in patients with CCC, data concerning the role of SBRT are scarce, and radiotherapy is restricted to patients who are not eligible for other treatments (6).

While liver function plays a significant role in patient stratification, the Child-Pugh (CP) classification incorporates serum albumin, bilirubin, and international normalized ratio (INR) and also the presence of hepatic encephalopathy and ascites for the evaluation, which are highly subjective and thus not easily reproducible, therefore leading to a significant detection bias with the result of under- or overestimation of liver function (10). Thus, the albumin–bilirubin (ALBI) grade was introduced as a more objective method to evaluate liver function (11) but has not been evaluated longitudinally in patients with primary liver cancer treated with SBRT within prospective trials. Since SBRT can lead to radiation-induced liver disease, a validated baseline assessment of liver function is critical for the accurate evaluation of candidates for SBRT.

In this study, we evaluated the role of SBRT in patients with locally advanced HCC or CCC treated within two consecutive prospective trials conducted in our institution (LAPIS trial and Heracles trial) with a focus on liver function.

## Materials and methods

### Study population

Study protocols and patient consent forms were approved by the institutional ethics committee (EK 374/15 and 38/16). Both trials were registered at the German Clinical Trials Register (Lapis trial, DRKS 00008566; Heracles trial, DRKS00011266). Patients unsuitable for any other local or systemic treatments or who progressed under local or systemic treatments were eligible for both trials after a multidisciplinary tumor board discussion. Inclusion and exclusion criteria are provided in the **Supplementary Material**.

### Treatment

Treatment planning and delivery have been also described in detail elsewhere (7, 12, 13). Patients were immobilized using a customized vacuum cushion, patient positioning boards, knee cushions, and abdominal compression and received a 4D and multiphase CT (arterial phase and/or delayed phase and venous phase). The gross tumor volume (GTV) was defined as the primary tumor and the tumor vascular thrombus if present. Image-guided radiotherapy (IGRT) was mandatory for every fraction. At-risk adopted dose prescription was applied considering the constraints for the organs at risk (OARs), aiming at a biologically effective dose (BED) of up to approximately 100 Gy depending on the proximity of the organs at risk. Dose prescription was chosen so that 95% of the planning target volume (PTV) received at least the nominal fraction dose and that 99% of the PTV received a minimum of 90% of the nominal dose [according to the International Commission on Radiation Units and Measurements (ICRU) 83, 91]. The dose maximum within the PTV was 110%–120% of the prescribed dose. Patients with smaller tumors not abutting any OARs received 3 × 18.75 Gy to the D50% such that 95% of the PTV received a minimum of 45 Gy (3 × 15 Gy, 80% of the nominal dose) and a dose maximum between 110% and 120% (14). For tumors abutting/overlapping with organs at risk, fractionations such as 5 × 10 Gy or 8 × 7.5 Gy were chosen in order to achieve the constraints for the organs at risk, while ultra-central tumors received mostly 66 Gy in 12 fractions of 5.5 Gy. For lesions where dose constraints for the OARs according to Timmerman (15) could not be achieved, a simultaneous integrated protection (SIP) dose prescription was used as described elsewhere (16, 17) in order to avoid dose reduction for the entire PTV.

For analysis, the prescribed dose matrices were converted to BEDs and equieffective doses for 2-Gy fractions (EQD2), using the linear quadratic model (LQ) as previously described (18). BEDs in Gy (10) for tumors or Gy (3) for normal lungs were calculated and converted to equivalent doses in 2-Gy fractions [=normalized total dose (NTD)] and to estimated log cell kill (18). For liver toxicity, we assumed an α/β value of 2 Gy, as also performed in previous analyses (19).

### Liver function response and toxicity evaluation

The Child-Pugh score and the albumin–bilirubin grade were assessed as previously described (11, 20, 21) at baseline (prior to treatment) and at every subsequent follow-up. Clinical outcome was assessed at 6 weeks after SBRT and then every 3 months. The response was evaluated according to the international criteria proposed in the modified Response Evaluation Criteria in Solid Tumors (mRECIST) Guideline version 1.1 (22). Toxicity was scored according to the National Cancer Institute (NCI) Common Terminology Criteria for Adverse Events (CTCAE) v4.0 for adverse events. Response assessment, toxicity evaluation, and blood tests (complete blood counts and biochemical analysis including liver function) were repeated every 3 months.

### Statistics

Overall survival (OS) was calculated as the time from the start of treatment until death from any cause, with censoring at the date last seen alive. Progression-free survival (PFS) was calculated as the time from the start of treatment until the last documented response assessment. OS and PFS were estimated using the Kaplan–Meier method. Continuous variables are presented as median with the corresponding range (minimum and maximum) and categorical variables as absolute and relative frequencies unless stated otherwise. The Cox proportional hazards regression model was used for further analyses of possible prognostic factors for OS and PFS and the logistic regression for the development of toxicities. The small number of patients did not allow complex multivariate modeling with variable selection using forward selection. Therefore, variables considered in a multivariate model were selected according to relevant univariate impact on OS or PFS. A forward variable selection approach was then applied. Local control (LC) was defined as the time from the start of SBRT until the progression of the treated lesion. Patients without progression were censored at the earlier of the last response assessment. Analyses for LC were conducted at the lesion level.

Death was considered a competing event. Analyses were performed using the SPSS software (IBM, SPSS, v27).

## Results

### Patient and treatment characteristics

Between 06/2016 and 06/2017, 83 patients were included in both prospective trials. A total of 20 patients were planned for SBRT in the Heracles trial, of whom two patients were excluded due to disease progression, resulting in 18 evaluable patients. In the LAPIS trial, 63 patients were included, of whom 12 were excluded either due to disease progression or alternative treatments, resulting in a total of 69 evaluable patients with 74 SBRT-treated liver lesions. Patients had HCC in 84% of the cases and CCC in 16%. Most patients had an underlying liver disease (58%) and were pre-treated (49%). Nine patients (13%) had metastatic disease, and 16% had a portal vein tumor thrombus. Patient and treatment characteristics are shown in **Table 1**.

**Table 1:** Patient and treatment characteristics.

	Number of patients (% or range)
Age	
** Median (range)**	74 (53–93)
Gender	
** Male** ** Female**	54 (78%) 15 (22%)
**Hepatocellular carcinoma**	58 (84%)
**Cholangiocarcinoma**	11 (16%)
Etiology of liver disease	
** HBV** ** HCV**	7 (10%) 10 (15%)
**Alcohol induced**	16 (23%)
**NASH**	7 (10%)
Treatments before study inclusion†	
** No** ** Yes** ** Resection** ** RFA** ** TACE** ** SBRT** ** Systemic treatment** ** SIRT**	35 (51%) 34 (49%) 15 (21%) 3 (4%) 24 (35%) 5 (7%) 1 (1%) 7 (10%)
BCLC	
** A** ** B** ** C** ** D**	25 (36%) 24 (35%) 19 (28%) 1 (2%)
**Metastatic disease**	9 (13%)
Child-Pugh score baseline	
** A** ** B** ** C**	52 (76%) 16 (23%) 1 (1%)
ALBI grade	
** 1** ** 2** ** 3**	37(54%) 28 (41%) 4 (6%)
**ALBI score**	−2.56 (−1.01, −3.78)
**Albumin**	4 (2.6–5)
**Bilirubin**	0.8 (0.2–5.0)
**Maximal tumor diameter (median, range), mm**	48 (14–215)
**Portal vein thrombosis (PVT)**	16 (23%)
Total prescribed dose, median (IQR)	60 (50–60) Gy
BED_10_, median (IQR)	102 (90–105) Gy
Dose per fraction, median (IQR)	8 (6.5–12) Gy
Number of fractions, median (IQR)	8 (6–12)

HCC, hepatocellular carcinoma; CCC, cholangiocarcinoma; NASH, non-alcoholic steatohepatitis; BCLC, Barcelona Clinic Liver Cancer; TACE, transarterial chemoembolization; SIRT, selective internal radiation therapy; PVT, portal vein thrombosis; SBRT, stereotactic body radiation therapy; ALBI, albumin–bilirubin; RFA, radiofrequency ablation; HBV, hepatitis B virus; HCV, hepatitis C virus.

†Some patients received multiple treatments.

### Overall survival, progression-free survival, and local progression

**Figure 1:**
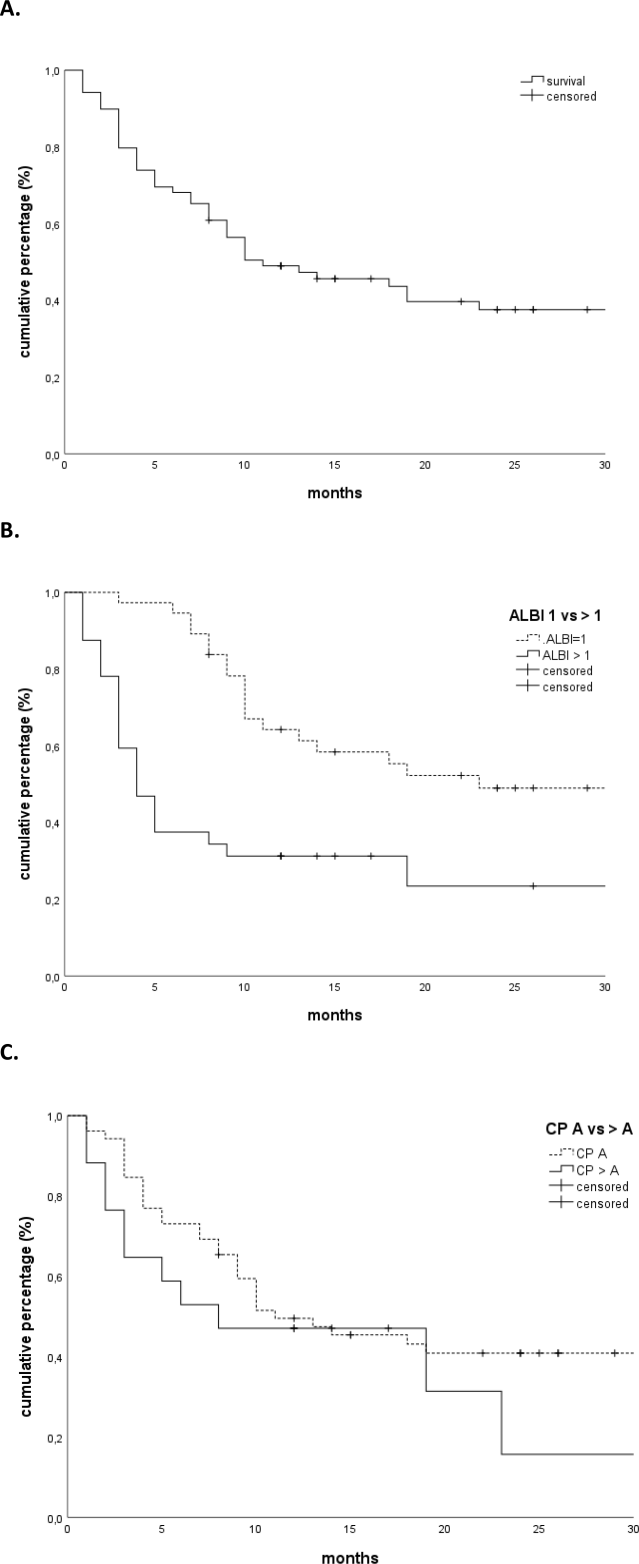
**(A)** Overall survival. **(B)** Overall survival stratified according to the ALBI grade (1 vs. >1). **(C)** Overall survival stratified according to Child-Pugh score (A vs. >A). ALBI, albumin–bilirubin.

The median follow-up was 29 months. The OS rates at 1 and 2 years were 49% and 38%, respectively, with a median OS of 11 months (**Figure 1A**). Patients with a higher CP score (HR 1.247, 95% CI 1.030–1.509, p = 0.02) and ALBI grade (HR 2.094, 95% CI 1.344–3.262, p = 0.001), larger tumors (HR 1.011, 95% CI 1.002–1.020, p = 0.02), and presence of portal vein thrombosis (PVT) (HR 2.137, 95% CI 1.094–4.173, p = 0.02) had a worse OS. Only the ALBI score remained significant in multivariate analysis (**Tables 2A**, **B**). Patients with an ALBI score of 1 (54%) had a median OS of 23 months (95% CI 3.148–42.852), while patients (46%) with an ALBI grade of ≥2 had a median OS of 4 months (95% CI 2.419–5.581, p < 0.001 log rank, **Figure 1B**). However, patients with a CP score of A had a median OS of 11 months versus 8 months for patients with a CP score of ≥B (p = 0.32 log rank, **Figure 1C**).

**Table 2A:** Univariate Cox regression analysis of overall survival (OS).

Parameter	HR	95% lower confidence limit	95% upper confidence limit	p
HCC vs. CCC	1.175	0.519	2.669	0.69
Child-Pugh score	1.247	1.030	1.509	0.02
ALBI grade	2.094	1.344	3.262	0.001
Portal vein thrombus	2.137	1.094	4.173	0.03
Tumor diameter	1.011	1.002	1.020	0.02
Prescribed dose (Gy)	0.997	0.964	1.031	0.87
BED (Gy)	1.001	0.999	1.003	0.39
Bilirubin (mg/dL)	0.963	0.720	1.287	0.79
Albumin (g/dL)	0.479	0.29	0.776	0.003

HCC, hepatocellular carcinoma; CCC, cholangiocarcinoma; ALBI, albumin–bilirubin; BED, biologically effective dose.

**Table 2B:** Multivariate Cox regression analysis of overall survival (OS).

Parameter	HR	95% lower confidence limit	95% upper confidence limit	p
ALBI grade	2.324	1.357	3.979	0.002
Portal vein thrombus	1.184	0.473	2.962	0.72
Tumor diameter	1.012	1.003	1.022	0.12

ALBI, albumin–bilirubin.

Four (6%) patients developed a local progression, of whom two had a regional progression in the liver at the same time. A total of 24 (35%) patients developed a regional regression, of whom five had distant metastases at the same time. In total, 16 patients (23%) developed distant metastases. The median PFS was 18 months. The PFS rates at 1 and 2 years were 53% and 38%, respectively. Patients with a PVT had a worse PFS (HR 2.659, 95% CI 1.271–5.549, p = 0.009, **Table 3**). The LC at 1 and 2 years was 91%. None of the clinical or treatment parameters correlated with the incidence of local tumor progression.

**Table 3:** Univariate Cox regression analysis of progression-free survival (PFS).

Parameter	HR	95% lower confidence limit	95% upper confidence limit	p
HCC vs. CCC	0.511	0.155	1.687	0.27
Child-Pugh score	1.023	0.805	1.300	0.85
ALBI grade	0.904	0.493	1.658	0.74
Portal vein thrombus	2.656	1.271	5.549	0.009
Tumor diameter	1.007	0.994	1.019	0.29
Prescribed dose (Gy)	1.084	0.974	1.208	0.14
BED (Gy)	0.997	0.989	1.005	0.47
Bilirubin (mg/dL)	0.945	0.706	1.266	0.71
Albumin (g/dL)	0.991	0.569	1.727	0.98

HCC, hepatocellular carcinoma; CCC, cholangiocarcinoma; ALBI, albumin–bilirubin; BED, biologically effective dose.

**Table 4:** Logistic regression analysis for the development of grade ≥ 3 hepatic failure.

Parameter	OR	95% lower confidence limit	95% upper confidence limit	p
HCC vs. CCC	0.674	0.070	5.567	0.64
Child-Pugh score	1.539	0.998	2.373	0.05
ALBI grade	6.136	1.664	22.626	0.006
Portal vein thrombus	1.769	0.388	8.061	0.46
Tumor diameter	1.018	0.998	1.040	0.08
Prescribed dose (Gy)	1.084	0.974	1.208	0.14
BED (Gy)	1.003	0.999	1.006	0.12
Bilirubin (mg/dL)	1.598	0.914	2.794	0.10
Albumin (g/dL)	0.323	0.100	1.050	0.06
Liver700cc* (EQD2, Gy)	1.050	0.983	1.123	0.15
Liver800cc** (EQD2, Gy)	1.036	0.992	1.081	0.11
Mean liver dose (EQD2, Gy)	0.982	0.902	1.069	0.67

HCC, hepatocellular carcinoma; CCC, cholangiocarcinoma; EQD2, equivalent dose at 2-Gy fractions; ALBI, albumin–bilirubin; BED, biologically effective dose.

*700 cc of uninvolved liver.

**800 cc of uninvolved liver.

### Toxicity

Thirteen patients developed grade ≥ 3 toxicities. Nine patients developed a hepatic failure, one patient developed a fistula, and three patients with pre-existing stents developed cholangitis. None of the patient characteristics for the dosimetric parameters correlated significantly with the development of grade 3 hepatic failure except for the Child-Pugh score (OR 1.539, 95% CI 0.998–2.373, p = 0.05) and the ALBI grade (OR 6.136, 95% CI 1.664–22.626, p = 0.006) (**Table 4**).

## Discussion

Over the past decades, there have been significant advances in the treatment of primary liver tumors. Although SBRT seems to lead to high rates of local control, overall survival prospective data are scarce. Furthermore, most prospective studies on SBRT include highly selected patients with a CP score of A or B7 and rarely report results using the ALBI score.

We show that patients with an ALBI score of 1 had a significantly better median overall survival (23 months, 95% CI 3.148–42.852) than patients with an ALBI grade of ≥2 (4 months, 95% CI 2.419–5.581, p < 0.001 log rank). However, the difference between the median overall survival in patients with a CP score of A (median OS of 11 months) compared to patients with a CP score of ≥B (median OS 8 months) was only moderate and not statistically significant (p = 0.32, log rank). Interestingly, none of the dosimetric parameters such as the mean liver dose or the dose or the low-dose region of the liver dose volume histogram curves (e.g., 800 cc or 700 cc) correlated with the incidence of toxicity. Our findings suggest that the ALBI score should be considered for treatment decisions in SBRT of HCC using a cut-off of 1, rather than the more subjective CP score that has been used previously. The ALBI score is a simple, evidence-based, objective, and discriminatory method of assessing liver function in HCC. It has been extensively tested in an international setting, unlike the CP score, which includes more subjective variables such as ascites and encephalopathy (11) and should be routinely calculated prior to treatment. These results should be validated in larger prospective trials, emphasizing patient stratification for SBRT depending on liver function.

In a recent meta-analysis (23) that reported long-term outcomes of SBRT for HCC and included 17 mostly retrospective studies, acute hepatic toxicity ≥ grade 3 ranged from 0% to 30%, but late toxicity was rare. The incidence of severe hepatic toxicity varied according to the criteria applied. Most studies included only patients with a CP score of A and/or up to B7. Importantly, the selection was based on the CP score and not on the most recent ALBI score. In addition, most studies were of retrospective design, thus limiting the evaluation of liver toxicity at follow-up and explaining the big range of reported acute hepatic toxicity ≥ grade 3. Indeed, it is a challenge to interpret liver toxicity in patients with HCC; thus, retrospective data without regular clinical evaluation and blood tests including liver parameters should be used with caution.

Previous smaller studies reported outcomes in patients with a CP score of B or C, showing a big difference in the median overall survival between patients treated with SBRT with a CP B7 score (median OS 10 months) versus patients with a CP score ≥ 8 (24). Andolino et al. (25) demonstrated that there is a relationship between the pretreatment CP score and the development of any type of toxicity (p = 0.035) as well as an increase of more than one grade in hematologic or hepatic dysfunction (p = 0.008). In a study by Lee et al. [17], 17% of patients had an increase in CP score of more than 2 points at 6 months. This suggests that patients with a CP score of A of B7 can be safely offered SBRT for HCC (26). Nevertheless, an ALBI grade of 1 does not correspond to a Child-Pugh score of A. In patients with a CP score of A, Murray et al. (27) showed in a retrospective study that the baseline ALBI score was superior compared to the CP score in predicting OS and toxicity. Indeed, they showed that patients with ALBI scores of 1 and 2 had a median OS of 19.8 and 8.5 months, respectively (p = 0.008), while CP-A5 and CP-A6 patients had median survivals of 17.5 and 10.4 months, respectively (p = 0.061), which is similar to our results. In the study by Jackson et al., ALBI-centric models performed similarly to indocyanine green retention (ICG)-centric models on multivariate analyses predicting toxicity (28).

Our study has several limitations such as the small sample size, potential selection bias, and the heterogeneous patient population regarding the baseline liver function and the dose per fraction. Another limitation of the study may be the inclusion of patients with different primary liver tumor histologies, as HCC tends to develop on the basis of an inflamed cirrhotic liver contrary to CCC and thus has the tendency to have higher CP or ALBI scores. We tried to address this point in the univariate analysis showing no statistical differences between the two histologies, although there were only a small number of patients with CCC, making the results not easy to interpret especially concerning patients with CCC. Nevertheless, this heterogeneity provided additional information concerning the sensitivity of the liver to radiotherapy. Additionally, this analysis was based on two consecutive prospective studies, providing important aspects concerning liver function and toxicity.

In conclusion, our results suggest that patient selection with adequate liver function based on the ALBI score may minimize SBRT-related liver toxicity. Our findings indicate that the ALBI score should be considered for treatment decision in SBRT of HCC using a cut-off of 1, rather than the more subjective CP score. These results should be validated in larger prospective trials, emphasizing patient stratification for SBRT depending on liver function. Patients with adequate liver function had significantly longer overall survival, while neither tumor characteristics nor dosimetric parameters correlated with the development of toxicity of SBRT.

## Data Availability

The original contributions presented in the study are included in the article/**Supplementary Material**. Further inquiries can be directed to the corresponding author.
