# Supplementary material for: The role of ALBI score in patients treated with stereotactic body radiotherapy for locally advanced primary liver tumors: a pooled analysis of two prospective studies
Source: Front Oncol. 2024 Oct 3;14:1427332. doi: 10.3389/fonc.2024.1427332 (PMC11484445; doi:10.3389/fonc.2024.1427332)
Supplement: Supplementary file 1 [file Table1.docx]

Supp. Table 1- Inclusion/Exclusion Criteria

| Inclusion Criteria |
| --- |
| Age ≥ 18 years, male and female  Histologically or cytologically confirmed hepatocellular carcinoma OR diagnosis made with characteristic enhancement in 4-Phase CT or MRI corresponding to AASLD- / EASL guidelines in cirrhotic patients.  Discussion in a routine multidisciplinary tumour board  Patients unsuitable for surgery, TACE*, RFA, or alcohol ablation  Understanding of procedure, significance and consequences of the study  Signed informed consent  Bilirubin has to be < 4 x the upper limit of normal, AST or ALT < 6 x the upper limit of normal, international normalized ratio < 1.5 except if patients are on oral anticoagulation, haemoglobin≥ 90 g/L, platelets ≥ 50 x 10^9^/L, and neutrophils ≥ 1.0 x 10^9^/L |
| Exclusion Criteria |
| None of the following criteria must be present at the time of registration:  Active hepatitis  (Child-Turcotte-Pugh (CTP) C liver score*)  (Hepatic encephalopathy more than Grade 1 according to Child Pugh criteria*)  Gastric, duodenal or variceal bleed within 2 months of registration  Prior radiotherapy of the region to be treated  For female patients: Pregnancy, planned pregnancy |

*this applies only for the first study. In the subsequent study, no limitations were applied.
